# Long-term outcomes of adjuvant radiotherapy after surgical resection of central neurocytoma

**DOI:** 10.1186/s13014-014-0242-2

**Published:** 2014-11-06

**Authors:** Yi-Dong Chen, Wen-Bin Li, Jin Feng, Xiao-Guang Qiu

**Affiliations:** Capital Medical University Cancer Center, Beijing Shijitan Hospital, Capital Medical University, Beijing, China; Department of Radiation Oncology, Beijing Tiantan Hospital, Capital Medical University, Beijing, China; Department of Radiation Oncology, Beijing Puren Hospital, Beijing, China

**Keywords:** Central neurocytoma, Radiotherapy, Toxicity, Resection, Adjuvant therapy

## Abstract

**Background and purpose:**

The role of adjuvant radiotherapy for central neurocytomas (CNs) is not clear. Therefore, we aimed to examine the clinical outcomes of treating histologically confirmed CNs with adjuvant RT after surgical resection.

**Material and methods:**

Sixty-three CN patients were retrospectively evaluated: 24 patients underwent gross total resection (GTR); 28, subtotal resection (STR); 9, partial resection (PR), and 2, biopsy (Bx). They underwent adjuvant RT after surgery (median dose, 54 Gy).

**Results:**

The median follow-up was 69 months (15–129 months). The 5-year overall survival (OS) and 5-year progression-free survival (PFS) were 94.4% and 95% after GTR + RT, 96.4% and 100% after STR + RT, and 100% and 90.9% after PR + RT. Only three patients had tumor recurrence: at the primary site at 30 and 24 months in two GTR + PR patients, and dissemination to the spinal cord at 75 months in one STR + RT patient. Thirty-eight (63.3%) patients experienced late neurotoxicity (28, grade 1; 7, grade 2; 3, grade 3). Short-term memory impairment was the most common toxicity.

**Conclusions:**

RT after incomplete resection (IR) led to OS and PFS comparable to those for GTR. Considering the excellent outcomes and limited late toxicity, adjuvant RT maybe a good option for CN patients who undergo IR.

## Introduction

Central neurocytomas (CNs) of World Health Organization (WHO) grade II [1] are uncommon tumors of the central nervous system and represent approximately 0.25–0.5% of all intracranial tumors. CN was described for the first time in 1982 by Hassoun and co-workers, and, by the early 1990s, it had become a well-defined clinical and pathological entity [2]. CNs are typically located supratentorially in the lateral ventricle(s) and/or the third ventricle. CN used to be considered a benign tumor due to the excellent clinical outcomes after treatment. However, long-term follow-up showed that CNs had a higher recurrence rate than expected, even after complete resection [3].

Because of the rarity of this tumor, no randomized clinical trials on CNs have been performed. Treatment strategies have been based on case reports and retrospective studies on small populations [4-6]. Surgery is the mainstay of treatment, and the extent of surgery is the most important prognostic factor affecting clinical outcomes [7]. In addition, radiotherapy (RT) can successfully control residual tumor after incomplete resection or recurrence [3,8]. It is important to investigate the role of adjuvant RT after resection, given the dismal local control rates in CN patients who undergo incomplete resection (IR). For example, in a study of 45 CN patients [3], one-third of the patients developed recurrence after gross tumor resection (GTR). The recurrence rates were even worse in patients who underwent IR. Rades et al. [9] conducted a meta-analysis of all the published data since 1982 from 91 centers and reported that the 10-year local control rate was only 35% in the 109 CN patients who received IR. The role of adjuvant RT after resection remains controversial, and the standard treatment for this tumor has not yet been established [7,9-14]. The main concern about the use of adjuvant RT in CN patients is that it may increase the risk of late neurotoxicity in these patients [4,5]. However, neurotoxicity of RT in patients with CN has not been reported till now, and the concerns about radiotoxicity mainly come from the long-term toxicity observed in cases of other low-grade primary cerebral neoplasms like glioma [15].

Here, we retrospectively investigated the long-term survival and late toxicities associated with adjuvant RT for neurocytoma; we hope that these findings will help guide oncologists in treatment decisions in such cases in the future.

## Methods and materials

### Patient population

Sixty-three patients with CN underwent surgical resection between January 2001 and October 2010 at Beijing Tiantan Hospital. All the patients received adjuvant RT after surgical resection at Beijing Tiantan Hospital, Beijing Shijitan Hospital or Beijing Puren Hospital. Institutional review board approval was obtained at these hospitals for retrospective review of the data. In all the patients, the CN diagnosis was confirmed by pathological tests. The dates of patients’ follow-up appointments were obtained through a review of hospital records, telephone interviews, and patient consultations.

### Treatment

Of the 63 patients with CN, 24 underwent gross total resection (GTR); 28, subtotal resection (STR); 9, partial resection (PR); and 2, biopsy only (Bx). The extent of resection was assessed by contrast-enhanced MR within 2 weeks after surgery. Based on the surgeon’s intraoperative observation and the MR image, the extent of resection was classified as GTR (absence of a macroscopically visible residue), STR (less than 10% residue remaining) or PR (10–50% residue remaining). Patient selection for postoperative RT was mostly based on the surgeon’s discretion. Because there is no standard management procedure for this rare malignant tumor, a majority of surgeons at Tiantan Hospital preferred adjuvant RT to decrease the chances of recurrence. From the 63 patients, 38 (60.3%) were treated with conventional focal RT, and 25 (39.7%) with three-dimensional conformal RT (3D-CRT, n = 18) or intensity-modulated radiation therapy (IMRT, n = 7). Simulation was performed generally a month after surgical resection. The median interval between surgery and RT was 47 days (range, 13 to 160 days).

Tumor volume is best defined using pre- and postoperative imaging. The planning target volume (PTV) represented a 3-mm geometric expansion of the clinical target volume, which was an anatomically constrained 10-mm expansion of the gross-residual tumor and/or tumor bed. In the whole series, the median total dose was 54 Gy (range, 46–60 Gy), and it was delivered in 1.8–2 Gy daily fractions. All patients completed RT according to the schedule.

### Follow-up

Follow-up ended on September 1, 2012. Fifty-two (82.5%, 52/63) patients underwent contrast-enhanced MRI of the brain every year after radiation, while eleven (17.5%, 11/63) patients discontinued the MRI scans after 1 to 4 years of follow-up. The median follow-up was 69 months, and the mean follow-up was 66 months (range, 15–129 months). Overall survival (OS) was calculated from the time of surgery until death or last follow-up, and progression-free survival (PFS) was calculated from the time of surgery until tumor recurrence or last follow-up. The date of progression was confirmed retrospectively based on the findings from the MRI scans or based on symptomatic deterioration consistent with tumor progression. Toxicity was scored retrospectively using the National Cancer Institute Common Terminology Criteria for Adverse Events version 4.0 [16].

### Statistical analysis

The Kaplan-Meier method was used to estimate the time from surgery to relapse and death, and the log-rank test was used to compare survival between groups. All analyses were carried out using the SPSS 17.0 statistical software, and p values less than 0.05 were considered statistically significant.

## Results

### Patient characteristics

The patient characteristics are shown in Table 1. The majority of patients presented with symptoms of increased intracranial pressure, including headache (43 cases, 67.2%) and nausea and vomiting (15 cases, 23.4%). The other symptoms included visual disturbance (17.2%), seizure (1.6%), dizziness (6.4%), sensory disturbance (4.8%), and others (6.4%). The involved areas included the left lateral ventricle (47.6%), right lateral ventricle (39.7%), and both lateral ventricles (12.7%). The tumors extended into the third ventricle in three cases. The median Karnofsky score was 90 (range, 30–100) before radiation. There were more patients with younger age (<28 years) in the GTR + RT group (18/24, 75%) than other groups (p = 0.005). Other factors including gender (p = 0.288) and tumor location (p = 0.984) were evenly distributed between groups.

**Table 1:** **Patient characteristics**

	**Value**	**%**
Gender (n)		
Male	37	58.8
Female	26	41.2
Age (y)		
Median	28	
Range	6–66	
Symptoms (n)		
Headache	43	67.2
Nausea and vomiting	15	23.4
Visual disturbance	11	17.2
Others	12	18.8
Tumor location (n)		
Left lateral ventricle	30	47.6
Right lateral ventricle	25	39.7
Both lateral ventricle	8	12.7
Initial therapy (n)		
GTR + RT	24	38.1
STR + RT	28	45.9
PR + RT	9	14.3
Bx + RT	2	3.2

*Abbreviations*: *GTR* gross total resection, *STR* subtotal resection, *PR* partial resection, *Bx* biopsy, *RT* radiotherapy.

### Survival

**Figure 1:**
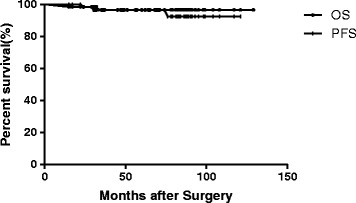
**Overall survival and progression-free survival in all the patients.**

**Figure 2:**
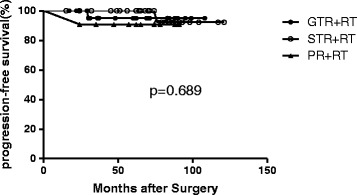
**Progression-free survival in the three groups: no statistically significant differences were observed between the three groups.**

Among the 63 CN patients, 42 patients were alive with no evidence of disease, and 8 were alive with stable disease. Eleven patients did not undergo MRI in recent years but had no symptoms of recurrence. Only two patients have died to date: one of them had undergone GTR and adjuvant RT and died due to tumor recurrence, while the other had undergone STR and adjuvant RT and died of unrelated causes 15 months after surgery. The 5-year OS was 96.6% and the 5-year PFS was 96.5% in all the patients (Figure 1). The 5-year OS and 5-year PFS were 94.4% and 95% respectively after GTR + RT, 96.4% and 100% respectively after STR + RT, and 100% and 90.9% respectively after PR + RT. No statistically significant differences were observed between the three groups with regard to the 5-year OS (p = 0.787) or 5-year PFS (p = 0.689) (Figure 2). The other factors including age, tumor diameter (≤5 cm vs. >5 cm), Karnofsky Performance Scale (KPS) score before RT (KPS ≤70 vs. >70), total radiation dose (≤54 Gy vs. >54 Gy), radiation technique (conventional RT vs. 3D-CRT/IMRT) and tumor location(unilateral ventricle vs. both ventricles) had no impact on OS or PFS (P >0.05).

Two patients underwent stereotactic biopsy and RT with total doses of 58 Gy and 54 Gy, which resulted in tumor shrinkage. One underwent STR at 5 months after RT and survived for 65 months without tumor progression; the other patient survived for 91 months after RT and is still alive without tumor recurrence.

### Recurrence and salvage treatment

Three patients had recurrences during follow-up. Among them, two patients had recurrences at the primary site at 30 months and 24 months after GTR and adjuvant RT, and underwent salvage re-resection; one of them died of tumor progression 3 months after surgery and the other is still alive, at 129 months after surgery. The third patient underwent STR and adjuvant RT with a total dose of 56 Gy and had tumor dissemination to the spinal cord at 76 months after surgery. Salvage RT was performed on the recurrent lesion, and this patient is still alive with residual disease (Table 2). One of three patients combined with the third ventricle invasion had recurrence.

**Table 2:** **Characteristics of the three patients with tumor progression after surgery**

**Patient no.**	**Therapy**	**TTP (mo)**	**Recurrence site**	**Salvage treatment**	**TAP (mo)**	**Last known status**
1	GTR + RT (54 Gy)	30	Primary site	PR	3	Dead, with disease
2	PR + RT (54 Gy)	24	Primary site	GTR	129	Alive, no disease
3	STR + RT (54 Gy)	76	Dissemination (spinal cord)	RT	9	Alive, with disease

*Abbreviations:* *GTR* gross total resection, *PR* partial resection, *STR* subtotal resection, *TTP* time to progression, *TAP* time after progression.

### Toxicity

The data on toxicity were available for 60 patients. Among them, 38 (63.3%) patients experienced late neurotoxicity (grade 1, n = 28; grade 2, n = 7; grade 3, n = 3). Short-term memory impairment was the most common toxicity. At the time of follow-up, 28 patients (28/60, 46.7%) had grade 1 short-term memory impairment, two of whom also had grade 1 motor deficit. Grade 2 neurotoxicity (7/60, 11.7%) included cognitive disturbance (n = 3), motor deficit (n = 3), seizure (n = 1), involuntary movements (n = 1) and hemianopsia (n = 1); although these conditions affected the activities of daily living of the affected patients, they did not require any assistance in their daily life. Only three patients (3/60, 5%) had grade 3 late neurotoxicity, including decreased vision (n = 1) and severe cognitive disability (n = 2). The first patient developed severe visual disturbance after partial resection and now requires assistance with his daily activities. The second patient had hydrocephalus and intracranial infection 4 weeks after surgery, and consequently developed irreversible grade 3 cognitive disturbance. The third patient developed cerebral infarction 5 years after RT, and had unilateral limb weakness and severe cognitive deficit at the time of follow-up.

Dosimetric comparison was performed in 25 patients who were treated with modern radiation techniques (18 with 3D-CRT and 7 with IMRT); the mean and maximum doses for organs at risk (OARs) were not significantly different between the group that exhibited neurotoxicity and the group that did not (Table 3). The hippocampus had not been delineated as an OAR in the previous treatment plans, so we re-planned the dosimetric comparison in order to add the hippocampus as an OAR. No significant difference was observed between the group that showed memory impairment and the group that did not, with regard to the mean and maximum radiation doses delivered to the hippocampus (p >0.05).

**Table 3:** **Mean and max doses for PTVs and OARs in the group with neurotoxicities versus the group with no neurotoxicities**

		**Neurotoxicity**		**No neurotoxicity**		**p**
		**Dose (cGy)**	**SD**	**Dose (cGy)**	**SD**	
PTV	Max	5736	365	5670	394	0.641
	mean	5470	348	5420	390	0.553
Brainstem	max	4809	1618	4340	1393	0.837
	mean	1389	800	874	689	0.462
Optic nerve	max	198	188	155	120	0.755
	mean	126	89	98	77	0.539
Left len	max	131	204	96	189	0.764
	mean	52	28	34	30	0.781
Right len	max	51	18	34	22	0.540
	mean	45	15	29	19	0.458
Hippocampus	max	3665	1753	4185	1804	0.740
(both sides)	mean	1947	1058	1592	772	0.303

*Abbreviations*: *PTV* planning target volume, *OAR* organ at risk.

## Discussion

To the best of our knowledge, this is one of the largest original studies investigating the long-term survival, local control and toxicity outcomes of adjuvant RT for CN. To date, data on the long-term outcomes of RT have been limited (Table 4). One meta-analysis [9] did show that RT improved the 10-year OS and 10-year LC in patients who underwent IR; moreover, this study also showed that RT significantly improved the OS and LC in cases of both typical CN and atypical CN. However, the results of the published meta-analysis were limited by the fact that it was conducted on preexisting published data from many institutions. Our results suggested that the addition of adjuvant RT led to the comparable survival outcomes to those who underwent GTR.

**Table 4:** **Findings of some large retrospective studies on central neurocytomas**

**Author**	**Year**	**N**	**Treatment**	**OS**		**LC**		**F/U (median)**	**Progression**
				**5-year (%)**	**10-year (%)**	**5-year (%)**	**10-year (%)**		
Current study	2013	63	GTR + RT(24)	96.6	N/A	96.5	N/A	69 mo	GTR + RT(1/24)
			STR + RT(28)					(15–129)	STR + RT(1/28)
			PR + RT(9)						PR + RT(1/9)
			Bx + RT(2)						Bx + RT(0/2)
Vasiljevic et al. [7]	2012	71	GTR(43)	N/A	N/A	N/A	N/A	48 mo	GTR(4/43)
			STR(13)					(6–204)	STR(9/28)
			STR + RT(4)						
			STR + S(6)						
			STR + CT(2)						
Hallock et al. [13]	2011	19	GTR(10)	N/A	82	N/A	60	104.5 mo	GTR(1/10)
			STR(8)					(0.75–261.7)	STR(4/8)
			STR + RT(1)						STR + RT(1/1)
Leenstra et al. [3]	2007	45	GTR(15)	83	83	67	60	10 y	LR(15/45)
			STR(14)					(1.6–23.4)	GTR(5/15)
			GTR + RT(6)						
			STR + RT(8)						
			BX + RT(1)						
Lenzi et al. [17]	2006	20	GTR(10)	N/A	N/A	N/A	N/A	7 y (3–20)	GTR(1/10)
			STR + RT(2)						STR + RT(0/2)
			STR(1)						STR(0/1)
			PR(3)						PR(3/3)
			PR + RT(4)						PR + RT(3/4)
Rades et al. [9]	2006	438	GTR(152)	99	99	84	74	44 mo	N/A
			GTR + RT(43)	97	97	87	87	(12–456)	
			IR(109)	82	82	41	35		
			IR + RT(134)	89	89	83	76		

*Abbreviations*: *GTR* gross total resection, *STR* subtotal resection, *PR* partial resection, *Bx* biopsy, *OS* overall survival, *LC* local control, *LR* local recurrence, *N/A* not applicable, *F/U* follow-up, *IR* incomplete resection, *RT* radiotherapy.

In this study, only three patients had recurrences during follow-up. According to the published data, the extent of resection is the most important prognostic factor, local control is better after GTR than after IR [14]. In some studies, high MIB-1proliferation index is also a prognostic factor which is associated with poorer local control [18-20]. Radiation is important factor that can retard residual tumor growth and improve local control [14], but the effect of radiation dose to local control remained unclear and should be addressed. The local control rate in our study is excellent, even in patients who underwent IR and adjuvant RT. Tumor location seemed to be irrelevant to local recurrence, but involvement of periventricular parenchyma was associated with poor local control in some cases [21]. In our study, one of three patients with the lateral ventricle and the third ventricle involvement had recurrence. It is hard to draw conclusion from such limited dada, but we guess that the extent of tumor invasion may affect the local recurrence and deserve further investigation. Other factors such as age and gender may not correlate to local recurrence based on the published data.

Neurotoxicity of RT in patients with CN has not been reported till now. Although Hallock et al. [13] did report the first large study on toxicity outcomes in CN patients, mainly the sequelae of surgery, only one patient received adjuvant RT (1/19) in their study. Our results showed that surgery plus adjuvant RT produced minimal late toxicity: only seven (11.7%) and three patients (5%) had grade 2 and 3 late toxicity, respectively, and no grade 4 and 5 late toxicity was observed. The grade 3 late toxicity was caused by surgery in two patients and by cerebral infarction 5 years after RT in the third patient. Although vasculopathy with stroke are rare sequelae of irradiation [22], it is still possible that they are related to adjuvant RT. Nonetheless, this is only a speculation, and this possible association should be studied further in future studies.

The most common late neurotoxicity was short-term memory impairment, which had no impact on the daily life and work of the affected patients. It is not clear whether this is related to RT, because the records of acute toxicity after surgery are incomplete. It has been reported that radiation doses to key structures such as the hippocampus may lead to long-term memory effects [23,24]; however, dosimetric comparison of the group with memory impairment and the group without memory impairment showed that the mean and maximum doses applied to the hippocampus did not differ significantly. Therefore, it seems that it is unlikely that RT was the main cause of the memory deficit. Instead, some studies [25,26] have shown that surgical resection has adverse effects on memory in patients in whom the tumor is located in the lateral ventricle and/or the third ventricle. Friedman et al. [26] retrospectively analyzed the neuropsychological data of 33 patients who received surgical treatment for brain tumors in the third ventricle: 43% of the patients displayed particularly severe memory impairment. Moreover, Shi et al. [27] showed that CN patients who underwent surgical resection showed impaired memory function in comparison with healthy individuals. In contrast, with regard to the effect of RT, most prospective studies [28-33] investigating radiation-induced neurotoxicity in low-grade glioma patients did not find a relationship between RT and cognitive impairment. Moreover, most studies that suggested a decline in cognitive function after RT were retrospective and often included a small number of patients [34]. Therefore, based on the published findings, we believe that the late neurotoxicities observed in our study were predominantly caused by surgery. Taken together, the findings in our study imply that the adverse effects of adjuvant RT for CN patients are limited and acceptable. Thus, in patients for whom it is difficult to perform GTR, IR with adjuvant RT avoids the potential risk of complications associated with a radical surgical approach.

Based on some studies, another reason for defering RT in patients who undergo IR is that they may be successfully salvaged with surgery or radiation in case of disease progression, these authors suggested that careful surveillance alone after IR may be a safe strategy [5,13]. However, other authors [9] argued that both OS and LC were important endpoints. At the time of recurrence, initially benign neurocytomas may be associated with severe complications such as craniospinal dissemination and intraventricular hemorrhage [35,36]. A second cranial operation appears to entail significant risk. Therefore, in our opinion, RT would be more effective if the tumor burden is minimal, and lower radiation dose and a limited radiation field are feasible at the time. The benefits of adjuvant RT seemed to overweigh its potential risks for CN patients after IR. Although it is hard to draw a conclusion based on the small sample size in this retrospective study, we believe that the role of adjuvant RT should be re-evaluated for these patients. Longer follow-up is needed to further investigate this issue.

For CN patients who undergo GTR, most authors agree that the clinical outcomes are favorable. Rades et al. [9] reviewed 152 CN cases of GTR and found the 5-year OS and 5-year LC to be 99% and 84%, respectively; these results are in accordance with ours (94.4% and 95%, respectively). All these data suggest that GTR alone is sufficient for the treatment of most CN patients.

In our study, two patients who underwent biopsy and RT (one underwent surgery 6 months after RT) survived for 65 and 91 months without tumor progression. Similar to these cases, Leenstra et al. [3] summarized a total of 11 cases in which the patients were treated with conventional RT alone after stereotactic biopsy; tumor relapse occurred in only three cases. These limited data suggest that RT may be the treatment of choice for CN patients who undergo biopsy and do not require resection.

The optimal target volume of adjuvant RT for CN remains unclear because the data for this are scarce. Following the generally accepted guidelines for low-grade brain tumors, many institutions adopt a target volume that includes the preoperative tumor volume and a 2-cm margin [37]. Since CNs are indolent tumors and are mostly confined to the lateral ventricles without invasion to the surrounding brain parenchyma, we think that the target volume maybe somewhat different from that of other low-grade brain tumors. In recent years, we have performed conformal irradiation with a 10-mm clinical target volume margin in order to reduce the adverse effects of RT. Although the follow-up time was relatively short, no recurrence was observed in our patients. In the most recent largest prospective study [33] addressing the late effects in 78 pediatric patients with low-grade glioma treated with 3D-CRT, a 10-mm clinical target volume margin was routinely used; the results showed that the adverse effects were limited and predictable for most patients. Moreover, the 10-year OS and 10-year event-free survival were 96% and 75%, respectively. According to these limited data, we think that a 10-mm clinical target volume margin may be sufficient for CN patients who undergo 3D-CRT.

The optimal radiation dose for CN patients also remains unclear. The study by Rades [37] is the only one that investigates the appropriate radiation dose after IR; according to this study, a dose of ≥54 Gy significantly improves local control in patients with subtotally resected neurocytomas. However, in contrast to these findings, in our study, most of the patients received a total dose of 50–54 Gy, and higher doses (≥54 Gy) were not found to have an impact on clinical outcomes. Thus, a total dose of 50–54 Gy may be appropriate for CN patients who undergo IR.

One of the limitations in our study is that the MIB-1 labeling index of CN was not routinely examined at Tiantan Hospital in the past. The MIB-1 labeling index for this tumor is usually low, less than 2%, and tumors with indices greater than 2% or 3% have been referred to as “atypical neurocytomas” and are associated with a significantly shorter recurrence-free interval [18-20]. However, a recent large multicenter study of 71 cases by Vasiljevic and co-workers [7] reported that histologic criteria may not be reliable markers for “atypical” CNs, and that the extent of surgery was the main prognostic factor. Further, the long-term clinical results of 58 CN patients also challenged previous reports that the MIB-1 labeling index is related to the risk of recurrence [38]. In light of these reports, it seems that more reliable criteria are needed to define atypical groups of CNs.

## Conclusions

In CN patients who undergo IR, the addition of adjuvant RT leads to survival outcomes comparable to those of patients who undergo GTR. The excellent outcomes and low incidence of late toxicities suggest that adjuvant RT may play an important role in the treatment of CN patients after IR, considering that treatment with IR alone may lead to disease progression. A multicenter prospective randomized trial is warranted to further investigate the optimal parameters for adjuvant RT in CN patients.

## References

[1] Figarella-Branger D, Söylemezoglu F, Burger PC, Louis DN[M], Ohgaki H, Wiestler OD, Cavenee WK (2007). Central neurocytoma and extraventricular neurocytoma. WHO Classification of Tumours of the Central Nervous System.

[2] Hassoun J, Söylemezoglu F, Gambarelli D, Figarella-Branger D, von Ammon K, Kleihues P (1993). Central neurocytoma: a synopsis of clinical and histological features. Brain Pathol.

[3] Leenstra JL, Rodriguez FJ, Frechette CM, Giannini C, Stafford SL, Pollock BE, Schild SE, Scheithauer BW, Jenkins RB, Buckner JC, Brown PD (2007). Central neurocytoma: management recommendations based on a 35-year experience. Int J Radiat Oncol Biol Phys.

[4] Kim DG, Paek SH, Kim IH, Chi JG, Jung HW, Han DH, Choi KS, Cho BK (1997). Central neurocytoma: the role of radiation therapy and long-term outcome. Cancer.

[5] Schild SE, Scheithauer BW, Haddock MG, Schiff D, Burger PC, Wong WW, Lyons MK (1997). Central neurocytomas. Cancer.

[6] Nakagawa K, Aoki Y, Sakata K, Sasaki Y, Matsutani M, Akanuma A (1993). Radiation therapy of well-differentiated neuroblastoma and central neurocytoma. Cancer.

[7] Vasiljevic A, François P, Loundou A, Fèvre-Montange M, Jouvet A, Roche PH, Figarella-Branger D (2012). Prognostic factors in central neurocytomas: a multicenter study of 71 cases. Am J Surg Pathol.

[8] Paek SH, Han JH, Kim JW, Park CK, Jung HW, Park SH, Kim IH, Kim DG (2008). Long-term outcome of conventional radiation therapy for central neurocytoma. J Neurooncol.

[9] Rades D, Schild SE (2006). Treatment recommendations for the various subgroups of neurocytomas. J Neurooncol.

[10] Patil AA, McComb RD, Gelber B, McConnell J, Sasse S (1990). Intraventricular neurocytoma: a report of two cases. Neurosurgery.

[11] Barbosa MD, Balsitis M, Jaspan T, Lowe J (1990). Intraventricular neurocytoma: a clinical and pathological study of three cases and review of the literature. Neurosurgery.

[12] Rades D, Fehlauer F (2002). Treatment options for central neurocytoma. Neurology.

[13] Hallock A, Hamilton B, Ang LC, Tay KY, Meygesi JF, Fisher BJ, Watling CJ, Macdonald DR, Bauman GS (2011). Neurocytomas: long-term experience of a single institution. Neuro Oncol.

[14] Rades D, Fehlauer F, Lamszus K, Schild SE, Hagel C, Westphal M, Alberti W (2005). Well-differentiated neurocytoma: what is the best available treatment?. Neuro Oncol.

[15] Douw L, Klein M, Fagel SS, van den Heuvel J, Taphoorn MJ, Aaronson NK, Postma TJ, Vandertop WP, Mooij JJ, Boerman RH, Beute GN, Sluimer JD, Slotman BJ, Reijneveld JC, Heimans JJ (2009). Cognitive and radiological effects of radiotherapy in patients with low-grade glioma: long-term follow-up. Lancet Neurol.

[16] Common Terminology Criteria for Adverse Events and Common Toxicity Criteria National Cancer Institute Cancer Therapy Evaluation Program; Available from:http://ctep.cancer.gov/protocolDevelopment/electronic_applications/ctc.htm Accessed October 10, 2012.

[17] Lenzi J, Salvati M, Raco A, Frati A, Piccirilli M, Delfini R (2006). Central neurocytoma: a novel appraisal of a polymorphic pathology. Our experience and a review of the literature. Neurosurg Rev.

[18] Christov C, Alde-Biassette H, Le Guerinel C (1999). Recurrent central neurocytoma with marked increase in MIB-1 labelling index. Br J Neurosurg.

[19] Rades D, Schild SE, Fehlauer F (2004). Prognostic value of the MIB-1 labeling index for central neurocytomas. Neurology.

[20] Sharma MC, Rathore A, Karak AK, Sarkar C (1998). A study of proliferative markers in central neurocytoma. Pathology.

[21] Kim DG, Kim JS, Chi JG, Park SH, Jung HW, Choi KS, Han DH (1996). Central neurocytoma: proliferative potential and biological behavior. J Neurosurg.

[22] Grill J, Couanet D, Cappelli C, Habrand JL, Rodriguez D, Sainte-Rose C, Kalifa C (1999). Radiation-induced cerebral vasculopathy in children with neurofibromatosis and optic pathway glioma. Ann Neurol.

[23] Gondi V, Hermann BP, Mehta MP, Tomé WA (2012). Hippocampal dosimetry predicts neurocognitive function impairment after fractionated stereotactic radiotherapy for benign or low-grade adult brain tumors. Int J Radiat Oncol Biol Phys.

[24] Gondi V, Tome WA, Marsh J, Struck A, Ghia A, Turian JV, Bentzen SM, Kuo JS, Khuntia D, Mehta MP (2010). Estimated risk of perihippocampal disease progression after hippocampal avoidance during whole-brain radiotherapy: safety profile for RTOG 0933. Radiother Oncol.

[25] D'Angelo VA, Galarza M, Catapano D, Monte V, Bisceglia M, Carosi I (2008). Lateral ventricle tumors: surgical strategies according to tumor origin and development–a series of 72 cases. Neurosurgery.

[26] Friedman MA, Meyers CA, Sawaya R (2008). Neuropsychological effects of third ventricle tumor surgery. Neurosurgery.

[27] Shi ZF, Sun DL, Song JP, Yao Y, Mao Y (2011). Emotion and cognitive function assessment of patients with central neurocytoma resection through transcortical frontal approach: a 5-year postoperative follow-up study. Chin Med J (Engl).

[28] Laack NN, Brown PD, Ivnik RJ, Furth AF, Ballman KV, Hammack JE, Arusell RM, Shaw EG, Buckner JC, North Central Cancer Treatment Group (2005). Cognitive function after radiotherapy for supratentorial low-grade glioma: a North Central Cancer Treatment Group prospective study. Int J Radiat Oncol Biol Phys.

[29] Vigliani MC, Sichez N, Poisson M, Delattre JY (1996). A prospective study of cognitive functions following conventional radiotherapy for supratentorial gliomas in young adults: 4-year results. Int J Radiat Oncol Biol Phys.

[30] Armstrong CL, Hunter JV, Ledakis GE, Cohen B, Tallent EM, Goldstein BH, Tochner Z, Lustig R, Judy KD, Pruitt A, Mollman JE, Stanczak EM, Jo MY, Than TL, Phillips P (2002). Late cognitive and radiographic changes related to radiotherapy: initial prospective findings. Neurology.

[31] Brown PD, Buckner JC, O'Fallon JR, Iturria NL, Brown CA, O'Neill BP, Scheithauer BW, Dinapoli RP, Arusell RM, Curran WJ, Abrams R, Shaw EG (2003). Effects of radiotherapy on cognitive function in patients with low-grade glioma measured by the folstein mini-mental state examination. J Clin Oncol.

[32] Torres IJ, Mundt AJ, Sweeney PJ, Llanes-Macy S, Dunaway L, Castillo M, Macdonald RL (2003). A longitudinal neuropsychological study of partial brain radiation in adults with brain tumors. Neurology.

[33] Merchant TE, Conklin HM, Wu S, Lustig RH, Xiong X (2009). Late effects of conformal radiation therapy for pediatric patients with low-grade glioma: prospective evaluation of cognitive, endocrine, and hearing deficits. J Clin Oncol.

[34] Scoccianti S, Detti B, Cipressi S, Iannalfi A, Franzese C, Biti G (2012). Changes in neurocognitive functioning and quality of life in adult patients with brain tumors treated with radiotherapy. J Neurooncol.

[35] Eng DY, DeMonte F, Ginsberg L, Fuller GN, Jaeckle K (1997). Craniospinal dissemination of central neurocytoma. Report of two cases. J Neurosurg.

[36] Jamshidi J, Izumoto S, Yoshimine T, Maruno M (2001). Central neurocytoma presenting with intratumoral hemorrhage. Neurosurg Rev.

[37] Rades D, Schild SE, Ikezaki K, Fehlauer F (2003). Defining the optimal dose of radiation after incomplete resection of central neurocytomas. Int J Radiat Oncol Biol Phys.

[38] Kim JW, Kim DG, Kim IK, Kim YH, Choi SH, Han JH, Park CK, Chung HT, Park SH, Paek SH, Jung HW (2013). Central neurocytoma: long-term outcomes of multimodal treatments and management strategies based on 30 years' experience in a single institute. Neurosurgery.

